# CircSETDB1 knockdown inhibits the malignant progression of serous ovarian cancer through miR-129-3p-dependent regulation of MAP3K3

**DOI:** 10.1186/s13048-021-00875-0

**Published:** 2021-11-17

**Authors:** Bo Li, Lu Zhang

**Affiliations:** Department of Gynaecology, Yantaishan Hospital, No.91 Jiefang Road, Zhifu DistrictShandong Province, Yantai, 264001 China

**Keywords:** SOC, circSETDB1, miR-129-3p, MAP3K3

## Abstract

**Background:**

Circular RNA (circRNA) is recently found to participate in the regulation of tumor progression, including ovarian cancer. However, the application of circRNA SET domain bifurcated histone lysine methyltransferase 1 (circSETDB1) as a therapeutic target in serous ovarian cancer (SOC) remains to be elucidated. Herein, circSETDB1 role in SOC malignant progression and underlying mechanism are revealed.

**Methods:**

The expression of circSETDB1, microRNA-129-3p (miR-129-3p) and mitogen-activated protein kinase kinase kinase 3 (MAP3K3) messenger RNA (mRNA) was detected by quantitative real-time polymerase chain reaction. Protein abundance was determined by western blot analysis. Cell proliferation, apoptosis, invasion and migration were demonstrated by cell counting kit-8 and 5-Ethynyl-29-deoxyuridine assays, flow cytometry analysis, transwell invasion assay and wound-healing assay, respectively. The interaction between miR-129-3p and circSETDB1 or MAP3K3 was predicted by online database, and identified by mechanism assays. The effect of circSETDB1 knockdown on tumor formation in vivo was unveiled by mouse model experiment.

**Results:**

CircSETDB1 and MAP3K3 expression were apparently upregulated, whereas miR-129-3p expression was downregulated in SOC tissues and cells in comparison with normal fallopian tube tissues or normal ovarian epithelial cells. CircSETDB1 knockdown inhibited cell proliferation, invasion and migration, but induced cell apoptosis in SOC cells. Additionally, miR-129-3p inhibitor impaired circSETDB1 silencing-mediated SOC malignant progression. MiR-129-3p repressed SOC cell processes via binding to MAP3K3. Furthermore, circSETDB1 knockdown suppressed tumor growth in vivo.

**Conclusion:**

CircSETDB1 silencing repressed SOC malignant progression through miR-129-3p/MAP3K3 pathway. This study supports circSETDB1 as a new therapeutic target for SOC.

**Supplementary Information:**

The online version contains supplementary material available at 10.1186/s13048-021-00875-0.

## Highlights

1. CircSETDB1 expression was increased in SOC tissues and cells.

2. CircSETDB1 silencing repressed the malignancy of SOC cells.

3. CircSETDB1 mediated SOC malignant progression by interacting with miR-129-3p.

4. MAP3K3 served as a target gene of miR-129-3p.

5. CircSETDB1 knockdown inhibited tumor formation in vivo.

**Supplementary Information:**

The online version contains supplementary material available at 10.1186/s13048-021-00875-0.

## Introduction

Ovarian cancer (OC) is a lethal gynecological malignancy that ranks the eighth in incidence and mortality among female cancers globally [34]. Serous OC (SOC) is the common subtype of OC with a high rate of metastasis into abdomen at advanced stage, accounting for almost 80% of OC deaths [3]. Platinum-based chemotherapy combined with surgical operation is thought to be the major therapeutic method [4]. The high mortality and relapse are largely attributed to the indistinct pathogenesis and lack of targeted treatment. Thus, the identification of novel therapeutic target that assists in repressing SOC progression is urgent for the improvement of SOC patient prognosis.

Circular RNA (circRNA) is an endogenous conserved noncoding RNA and is generated through back-splicing events as loop, expressing in a wide array of organisms [18]. 3’-5’ covalent closed-loop structure of circRNA makes it lengthen its half-life, which creates possibility for circRNA as a biomarker [25]. Lots of circRNAs consist of exons and mainly function at the post-transcriptional level [17]. CircRNA commonly represses microRNA (miRNA) activity via serving as a competing endogenous RNA, and further modulates gene expression [1, 14]. Studies in recent years have revealed the roles of circRNAs in the genesis of various cancers, such as gastric carcinoma [24], cervical cancer [5], prostate cancer [15] and gastrointestinal cancers [30]. Numerous researches revealed the presence of dysregulated circRNAs in OC tissues [11, 23], indicating the relevance of circRNAs in OC malignant progression. According to the previous studies, circRNAs, including circ_0049116 [11], circ_0000479 [37] and circ_0051240 [41], have been identified to act oncogenic roles in OC progression. On the contrary, circ_0000284 [35] and circ_101057 [27] were found to inhibit OC progression. Another circRNA, circRNA SET domain bifurcated histone lysine methyltransferase 1 (circSETDB1), is indicated to be negatively correlated with progression-free survival time of SOC cases [36], suggesting that circSETDB1 may be involved in SOC progression; however, the underlying mechanism remains to be established.

As a noncoding RNA, miRNA is an endogenous short molecule with 18–25 nucleotides [16]. It can cause mRNA degradation or the repression of protein translation through perfect or imperfect complementarity with target gene, and thereby modulates cellular biological processes [2]. Tumor-suppressing or oncogenic role of miRNA depends on the function of its targeted gene [22]. MiR-129-3p, a miRNA, has been explained to suppress SOC development [28]. Mitogen-activated protein kinase kinase kinase (MAP3K) 3 (MAP3K3), located on 17q, is a number of MAP3K family of serine/threonine kinases and upstream regulates MAPK pathway [6]. In the study of Zhang and Wang, MAP3K3 was found to promote SOC carcinogenic progression by interaction with miR-212-3p [40]. The above evidences show the importance of miR-129-3p and MAP3K3 in SOC malignant development.

Our previous data have predicted the interaction between miR-129-3p and circSETDB1 or MAP3K3 by online databases. Thus, this study was designed to reveal the role of circSETDB1 in SOC progression in cell model and mouse model assays, and to unveil whether circSETDB1 mediated SOC malignant progression by miR-129-3p/MAP3K3 axis.

## Materials and methods

### Patients and the Ethics Committee

Under the agreement of the Ethics Committee of Yantaishan hospital, 73 SOC tissues and 73 fallopian tube tissues were collected from SOC sufferers and healthy women, respectively, in Yantaishan hospital. The diagnosis of SOC tissues was confirmed by two pathologists based on the histological feature: solid masses of cells with slit-like fenestrations, the papillary, glandular or cribriform architecture of tumors, as well as large, hyperchromatic and pleomorphic nuclei. The cases signed the written informed consent before surgery. All obtained tissues were stored at -80˚C.

### Cell culture and Actinomycin D treatment

Procell (Wuhan, China) provided the ovarian cancer cells (A2780 and SKOV3), normal ovarian epithelial cells (IOSE-80) and human embryonic kidney cells (293 T). A2780, IOSE-80 and 293 T cells as well as SKOV3 cells were maintained in Dulbecco’s modified Eagle’s medium (DMEM; Gibco, Carlsbad, CA, USA) and McCoy’s 5a medium (Gibco), respectively, and cultured at 37˚C in a humid atmosphere with 5% CO_2_. For confirming the stability of circSETDB1, both A2780 and SKOV3 cells were co-cultured with 2 mg/L Actinomycin D (Abcam, Cambridge, MA, USA) for 0, 4, 8, 12 and 24 h, respectively. CircSETDB1 expression was then detected by quantitative real-time polymerase chain reaction (qRT-PCR) with SETDB1 expression as a control.

### Cell transfection

GENEWIZ (Suzhou, China) provided oligonucleotides, including small interfering RNAs (siRNAs) targeting circSETDB1 (si-circSETDB1#1, si-circSETDB1#2 and si-circSETDB1#3) and MAP3K3 (si-MAP3K3), siRNA negative control (si-NC), miR-129-3p mimic, mimic NC, miR-129-3p inhibitor and inhibitor NC. MAP3K3 overexpression vector (pcDNA-MAP3K3) was built using pcDNA 3.1( +) vector (pcDNA; EK-Bioscience, Shanghai, China). For cell transfection, the cells grown in 12-well plates were transfected with siRNAs (40 pmol), miR-129-3p mimic (50 nmol/L), miR-129-3p inhibitor (100 nmol/L), pcDNA-MAP3K3 (2.0 μg) using TurboFect reagent (Thermo Fisher, Waltham, MA, USA) according to the users’ instruction. The oligonucleotide sequences were displayed as following. Si-circSETDB1#1 5’-GTCCAACCCTGATGTCCAAGT-3’; si-circSETDB1#2 5’-CAGTCCAACCCTGATGTCCAA-3’; si-circSETDB1#3 5’-TCAGTCCAACCCTGATGTCCA-3’; miR-129-3p mimic 5’-AAGCCCUUACCCCAAAAAGCAU-3’; miR-129-3p inhibitor 5’-AUGCUUUUUGGGGUAAGGGCUU-3’; si-MAP3K3 5’-GGAGCACAAGGTGACAACAGTATTT-3’; si-NC 5’-GCTTAAACGCATAGTAGGACT-3’; mimic NC 5’-UUUGUACUACACAAAAGUACUG-3’ and inhibitor NC 5’-CAGUACUUUUGUGUAGUACAAA-3’.

### RNA extraction and qRT-PCR

RNA from the SOC tissues and cells was isolated as per the guidebook of RNAsimple kit (Tiangen, Beijing, China). cDNA was synthesized using a FastKing RT Kit (Tiangen) or miRNA synthesis kit (TaKaRa, Dalian, China). Quantification of circSETDB1, miR-129-3p and genomic DNA was conducted with a FastFire qPCR PreMix (Tiangen) and following primers. Data were analyzed with the 2^−∆∆Ct^ method, and glyceraldehyde 3-phosphate dehydrogenase (GAPDH) and U6 were employed for normalization. The sequences of sense and anti-sense primers were listed as below. CircSETDB1 5’-TCTCCTGTGAAGCCTGAA-3’ and 5’-TGTAGCCTGGATAGTTAGTTG-3’; SETDB1 5’-AAGCGTCAAGTGGCAGTA-3’ and 5’-CATCATAGAATTGGCGTGT-3’; miR-129-3p 5’-ACACTCCAGCTGGGAAGCCCTTACCCCAAA-3’ and 5’-TGGTGTCGTGGAGTCG-3’; MAP3K3 5’-GAGCGTGACCCGAAAGTA-3’ and 5’-GCAGAGTCTCGGAGGATGTT-3’; GAPDH 5’-GGTCACCAGGGCTGCTTT-3’ and 5’-GGAAGATGGTGATGGGATT-3’; U6 5’-TCGCTTCGGCAGCACATAT-3’ and 5’-ATTTGCGTGTCATCCTTGC-3’.

### Cell counting kit-8 (CCK-8) assay

For determining cell proliferation, a CCK-8 Kit (Solarbio, Beijing, China) was employed. In brief, cultured A2780 and SKOV3 cells were passaged in 96-well plates (5000 cells per well) for 16 h. Oligonucleotides (si-circSETDB1#1, si-circSETDB1#2, si-NC, miR-129-3p mimic, mimic NC, miR-129-3p inhibitor and inhibitor NC) and plasmids (pcDNA-MAP3K3 and pcDNA) were transfected into cells for 0, 24, 48 and 72 h, respectively, based on the defined purposes. After that, cell supernatant was discarded and 10 μL CCK-8 solution (Solarbio) was incubated with cells. Three hours later, samples were assessed using microplate reader (Thermo Fisher) with the wavelength at 450 nm.

### 5-Ethynyl-29-deoxyuridine (EDU) assay

EDU assay was further used to study cell proliferation. A part of DMEM and McCoy’s 5a media were co-cultured with 100 μL EDU (50 μM; Ribobio, Guangzhou, China) for 120 min to label EDU. In brief, A2780 and SKOV3 cells were seeded in 12-well plates (1 × 10^5^ cells per well) and transfected with obvious treatments. At 48 h after transfection, cells were passaged in 96-well plates (5000 cells per well) containing EDU labeling medium. Then, EDU assay was conducted with an EDU staining kit (Ribobio). For staining nucleus, 4’,6-Diamidino-2-Phenylindole (DAPI; Beyotime, Shanghai, China) was employed. Finally, staining results were observed using a fluorescence microscope (Olympus, Tokyo, Japan).

### Flow cytometry analysis

Annexin V-fluorescein isothiocyanate (Annexin V-FITC) apoptosis detection kit (Solarbio) was utilized in this assay. In short, transfected cells were harvested and eluted with phosphate buffer solution (PBS; Solarbio). Then, 1 mL Binding Buffer (Solarbio) was used to suspend cells. Cells were orderly incubated with 5 μL Annexin V-FITC (Solarbio) and 5 μL propidium iodide (PI; Solarbio) in dark. Cell apoptotic rate was determined after cells were analyzed with flow cytometry (Thermo Fisher).

### Transwell invasion assay

12-well transwell chambers (Costar, Shanghai, China) were firstly pre-coated with Matrigel (100 μL; BD Biosciences, San Jose, CA, USA) for overnight, and placed into 12-well plates. After performing cell transfection for 48 h, 1 × 10^5^ A2780 cells or SKOV3 cells were suspended in serum-free DMEM (Gibco) or McCoy’s 5a medium (Gibco) and then loaded in the upper chambers. The corresponding medium was mixed with 15% FBS (Gibco) and added into the lower chambers to serve as chemoattractants. Twenty-four hours later, the cells on the lower chambers were fixed with 4% paraformaldehyde (Sigma, St Louis, MO, USA) and stained with 0.1% crystal violet (Sigma). Invaded cells were counted under microscope (Olympus) with 100 × magnification.

### Wound-healing assay

A2780 and SKOV3 cells grown in 6-well plates (2 × 10^5^ cells per well) were transfected with plasmids or oligonucleotides. After cells reached almost 100% confluence, the gaps in the confluent cell layer were made using pipette tips (Baisai, Shanghai, China). Meanwhile, floated cells and debris were discarded by wash using PBS (Solarbio). Cell migratory ability was analyzed after calculating the width of the scratch gaps under microscope (Olympus) with 40 × magnification at 0 or 24 h.

### Western blot analysis

The tissue and cell samples were lysed using NP-40 buffer (Beyotime), and proteins were degenerated by heating. Then, an aliquot of 20 μg protein samples were electrophoresed on 10% bis–tris-acrylamide gel (Thermo Fisher), followed by the wet-transfer with the nitrocellulose membranes (Pall Life Science, Beijing, China). After blocking in skimmed milk (Solarbio), the membranes were probed with anti-matrix metalloprotein 9 (anti-MMP9; 1:1000; Affinity, Nanjing, China), anti-MMP2 (1:1000; Affinity), anti-MAP3K3 (1:1000; Affinity) and anti-GAPDH (1:8000; Affinity). After that, the secondary antibody against goat anti-rabbit IgG (1:5000; Affinity) was incubated with the membranes. The protein signals were detected with eyoECL Plus (Beyotime). GAPDH was employed to normalize protein abundance.

### Dual-luciferase reporter assay

The binding sites between miR-129-3p and circSETDB1 or MAP3K3 3’-untranslated region (3’UTR) were predicted through starbase online database (http://starbase.sysu.edu.cn/agoClipRNA.php?source=mRNA). The wild-type (wt) and mutant plasmids of circSETDB1 and MAP3K3 were provided by the GeneCopoeia (Guangzhou, China), and named as circSETDB1 wt, circSETDB1 mut, MAP3K3 3’UTR wt and MAP3K3 3’UTR mut, respectively. In short, circSETDB1 wt and MAP3K3 3’UTR wt were built by sub-cloning the sequences of circSETDB1 and MAP3K3 3’UTR containing the binding sites of miR-129-3p into pmirGLO vector (EK-Bioscience). The complementary sites of circSETDB1 and MAP3K3 with miR-129-3p were mutated with Yeasen Site-Directed Mutagenesis Kit (Shanghai, China) to construct circSETDB1 mut and MAP3K3 3’UTR mut. Next, the plasmids (100 ng) were transfected into the 293 T cells cultured in 48-well plates with miR-129-3p mimic (50 nmol/L) or mimic NC (50 nmol/L) as per the guidebook. Finally, fluorescence signal was detected with Dual-Lucy Assay Kit (Solarbio). Results were expressed as the ratio of *firefly* and *Renilla* luciferase activity.

### RNA immunoprecipitation (RIP) assay

Magna RNA immunoprecipitation kit (Millipore, Billerica, MA, USA) was conducted in this part. In brief, A2780 and SKOV3 cells were harvested and lysed with RIP lysis buffer (Millipore). The lysates were incubated with the magnetic beads (Millipore) coated with anti-argonaute2 (Anti-Ago2; Abcam) or anti-immunoglobulin G (Anti-IgG; Abcam).The beads were eluted, and miR-129-3p and circSETDB1 expression in the complex were detected by qRT-PCR. Partial lysates not incubated with magnetic beads were employed as controls (Input).

### Mouse model experiment

Mouse model experiment was carried out with the agreement of the Animal Care and Use Committee of Yantaishan hospital. Female BALB/c nude mice (4–6 weeks of age; *N* = 12) were purchased from Charles River (Beijing, China). Lentivirus-mediated small hairpin RNA against circSETDB1 (sh-circSETDB1) and corresponding negative control (sh-NC) were packaged by FulenGen (Guangzhou, China). These mice were averagely divided into 2 groups. The establishment of mouse model of SOC was performed with stable SKOV3 cells in which the circSETDB1 was reduced (sh-circSETDB1) or not (sh-NC). 5 × 10^6^ SKOV3 cells were intraperitoneally inoculated into nude mice. The dimension of neoplasms was monitored every 5 days until the 30th day after injection as per volume (mm^3^) = width^2^ × length × 0.5. Thereafter, the mice were killed and the neoplasms were dissected. A part of peritoneal nodules were kept for further analysis in expression and weight.

### Immunohistochemistry (IHC) assay

IHC analysis was conducted based on the methods previously described [26]. In brief, the 4-µm-thick tissues were fixed, dehydrated and embedded into paraffin. Then, the sections were deparaffinized and antigen was retrieved by heating. After immersing in Hydrogen Peroxide (Millipore) for 10 min, the sections were incubated with anti-MAP3K3 (1:100; Abcam), anti-MMP9 (1:200; Affinity), anti-MMP2 (1:200; Affinity) and anti-nuclear proliferation marker (anti-ki-67), followed by the incubation with goat anti-rabbit IgG (1:200; Affinity). Hematoxylin (Millipore) was employed for staining the sections, and images were captured under a microscope (Olympus).

### Statistical analysis

All data analysis was performed with SPSS software (IBM, Somers, NY, USA), image J software (NIH, Bethesda, MD, USA) and GraphPad Prism software (GraphPad Software, La Jolla, CA, USA), and results were shown as means ± standard deviations (SD). Spearman’s correlation test was used for the comparisons in Spearman correlation analysis. Two-tailed Student’s *t*-tests or Wilcoxon rank-sum test was carried out to compare the significant differences between the two groups. One-way analysis of variance (ANOVA) was employed for assessing *P* value between the multiple groups. *P* < 0.05 was considered as statistical significance.

## Results

### CircSETDB1 was highly expressed in SOC tissues and cells

**Fig. 1:**
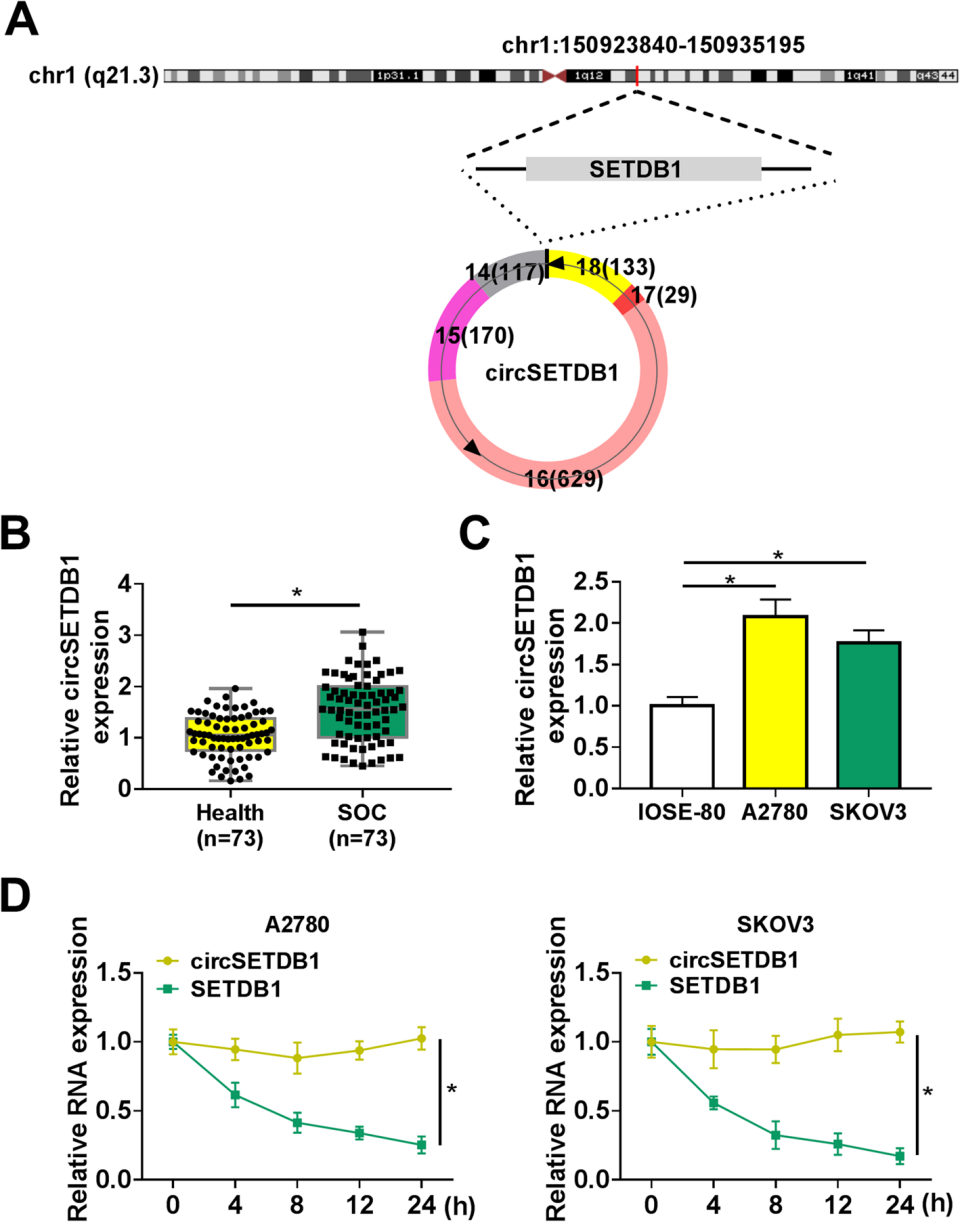
CircSETDB1 was overexpressed in SOC tissues and cells. **A** The schematic diagram demonstrated that circSETDB1 was generated from the exons 14–18 of SETDB1 through back-splicing. **B** and **C** CircSETDB1 expression was detected by qRT-PCR in SOC tissues (*N* = 73), normal fallopian tube tissues (*N* = 73) and IOSE-80, A2780 and SKOV3 cells. **D** The stability of circSETDB1 was determined by Actinomycin D treatment assay in A2780 and SKOV3 cells. *P < 0.05

CircSETDB1 was located in chromosome 1 (chr1):150923840–150935195, and was formed from the exons 14–18 of SETDB1 transcript by back-splicing (Fig. 1A). Its expression property was firstly determined in SOC tissues and cells to reveal its correlation with SOC progression. Results showed circSETDB1 was overexpressed in SOC tissues and cells (A2780 and SKOV3) when compared with normal fallopian tube tissues and IOSE-80 cells, respectively (Fig. 1B and C), which suggested circSETDB1 might be involved in SOC progression. Additionally, the stability of circSETDB1 was confirmed with Actinomycin D treatment assay. Results presented that circSETDB1 expression had no apparent change under Actinomycin D treatment, but the expression of its parent, SETDB1, was greatly downregulated (Fig. 1D), demonstrating that circSETDB1 was more stable than linear SETDB1.

### CircSETDB1 silencing repressed cell proliferation, invasion and migration, and induced cell apoptosis in A2780 and SKOV3 cells

**Fig. 2:**
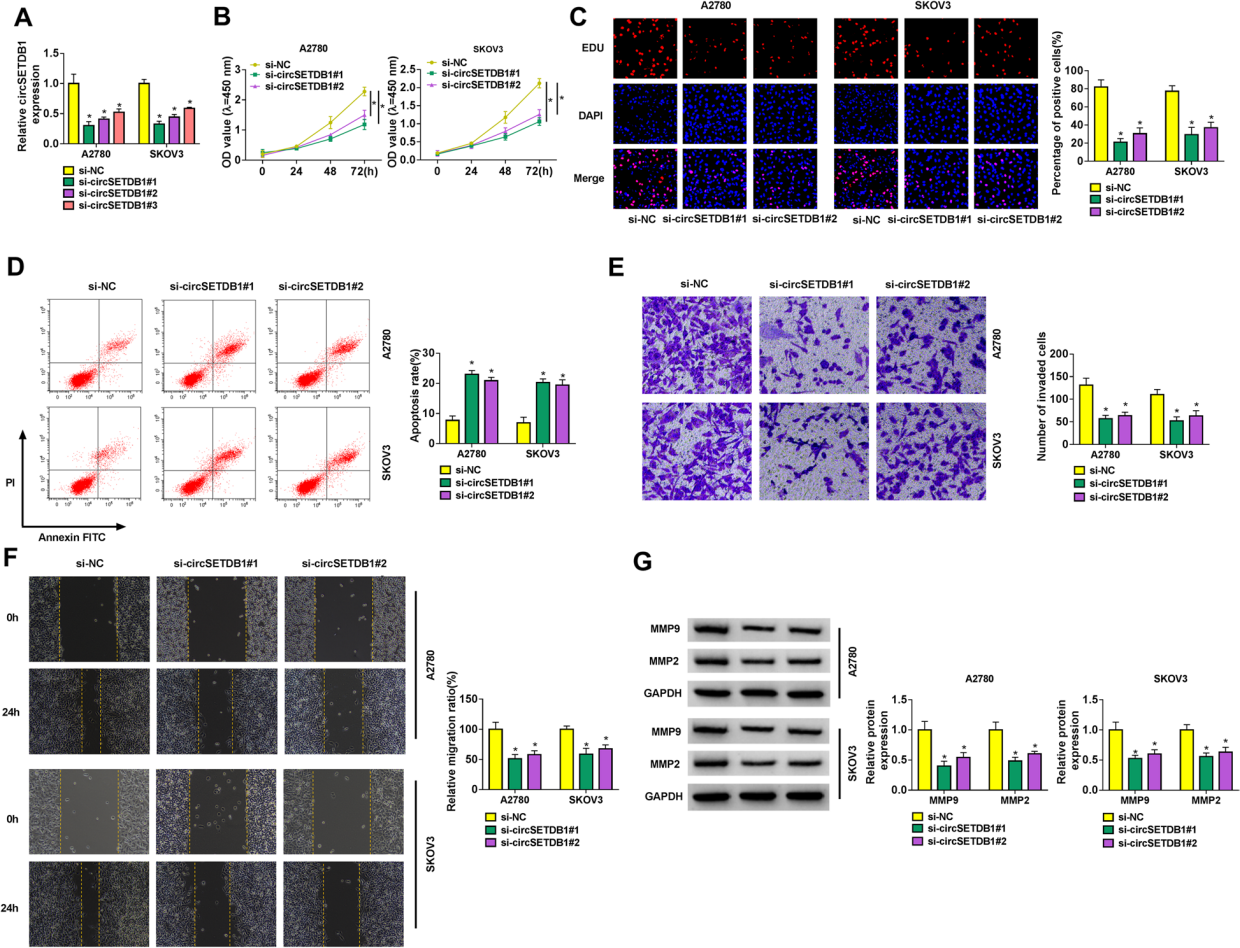
CircSETDB1 knockdown repressed SOC cell processes. **A** The interfering efficiency of si-circSETDB1#1, si-circSETDB1#2 and si-circSETDB1#3 was determined by qRT-PCR in A2780 and SKOV3 cells. **B** and **C** The effect of circSETDB1 knockdown on cell proliferation was revealed by CCK-8 and EDU assays in A2780 and SKOV3 cells. **D** The impact of circSETDB1 depletion on the apoptosis of A2780 and SKOV3 cells was determined by flow cytometry analysis. **E** and **F** Transwell invasion and wound-healing assays were employed to investigate the impacts of circSETDB1 downregulation on the invasion and migration of A2780 and SKOV3 cells, respectively. **G** Western blot analysis was performed to demonstrate the effect of circSETDB1 depletion on the protein abundance of MMP9 and MMP2 in A2780 and SKOV3 cells. *P < 0.05

To reveal the role of circSETDB1 in SOC cell processes, the interfering efficiency of si-circSETDB1#1, si-circSETDB1#2 and si-circSETDB1#3 was determined by qRT-PCR in A2780 and SKOV3 cells. Results showed that circSETDB1 expression was dramatically reduced by si-circSETDB1#1, si-circSETDB1#2 and si-circSETDB1#3 in A2780 and SKOV3 cells, especially by si-circSETDB1#1 and si-circSETDB1#2 (Fig. 2A). Based on the results from Fig. 2A, si-circSETDB1#1 and si-circSETDB1#2 were employed for further study. Subsequently, circSETDB1 knockdown repressed cell proliferation (Fig. 2B and C). On the contrary, cell apoptosis of A2780 and SKOV3 cells was induced after circSETDB1 silencing (Fig. 2D). Furthermore, the effects of circSETDB1 absence on the invasion and migration of A2780 and SKOV3 cells were unveiled. And data displayed that circSETDB1 depletion inhibited cell invasion and migration (Fig. 2E and F). In support, we observed downregulation of metastasis-related MMP9 and MMP2 expression after treatment of si-circSETDB1#1 or si-circSETDB1#2 using western blot analysis (Fig. 2G). Thus, the above results demonstrated that circSETDB1 might act as an oncogene in SOC malignant progression. Based on higher effect of si-circSETDB1#1 on SOC cell processes compared with si-circSETDB1#2, si-circSETDB1#1 was employed for subsequent study.

### CircSETDB1 functioned as a sponge for miR-129-3p in A2780 and SKOV3 cells

**Fig. 3:**
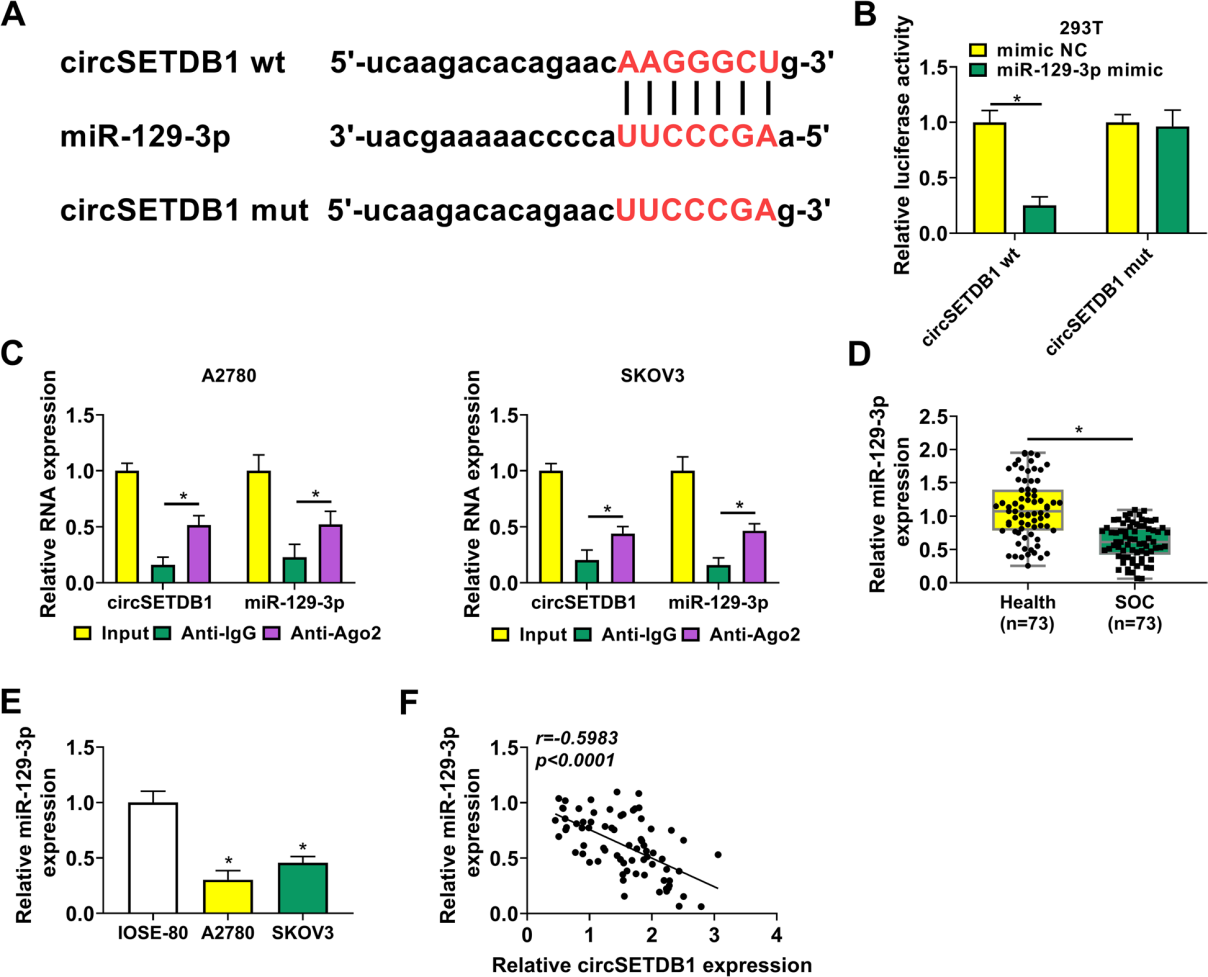
CircSETDB1 was associated with miR-129-3p in A2780 and SKOV3 cells. **A** Schematic illustration showed the putative binding sites of circSETDB1 in miR-129-3p and mutated sites of circSETDB1 sequence. **B** and **C** Dual-luciferase reporter and RIP assays were conducted to confirm the interaction between circSETDB1 and miR-129-3p in A2780 and SKOV3 cells. **D** and **E** MiR-129-3p expression was detected by qRT-PCR in SOC tissues (*N* = 73), normal fallopian tube tissues (*N* = 73) and IOSE-80, A2780 and SKOV3 cells. **F** The correlation between circSETDB1 and miR-129-3p expression was determined by Spearman correlation analysis in SOC tissues. *P < 0.05

The underneath mechanism by which circSETDB1 regulated SOC cell carcinogenesis was further revealed. To this purpose, we sought the circSETDB1-related miRNA. MiR-129-3p was found to contain the binding sites of circSETDB1 (Fig. 3A), and was employed as a candidate. Then, we found that luciferase activity was dramatically repressed in 293 T cells co-transfected with circSETDB1 wt and miR-129-3p mimic, but not in the cells co-transfected with circSETDB1 mut and miR-129-3p mimic (Fig. 3B). Also, RIP assay presented that both circSETDB1 and miR-129-3p expression were dramatically upregulated in Anti-Ago2 group as compared with them in Anti-IgG group (Fig. 3C). These results suggested that circSETDB1 was associated with miR-129-3p in SOC cells. Afterwards, dramatic downregulation of miR-129-3p was observed in SOC tissues and cells (A2780 and SKOV3) in comparison with normal fallopian tube tissues or IOSE-80 cells (Fig. 3D and E). When further studying the correlation between circSETDB1 and miR-129-3p in expression in SOC tissues, we found circSETDB1 was negatively related to miR-129-3p (Fig. 3F). These results meant that miR-129-3p might participate in circSETDB1-mediated SOC cell malignancy through interaction with circSETDB1.

### CircSETDB1 knockdown repressed SOC cell processes via binding to miR-129-3p

**Fig. 4:**
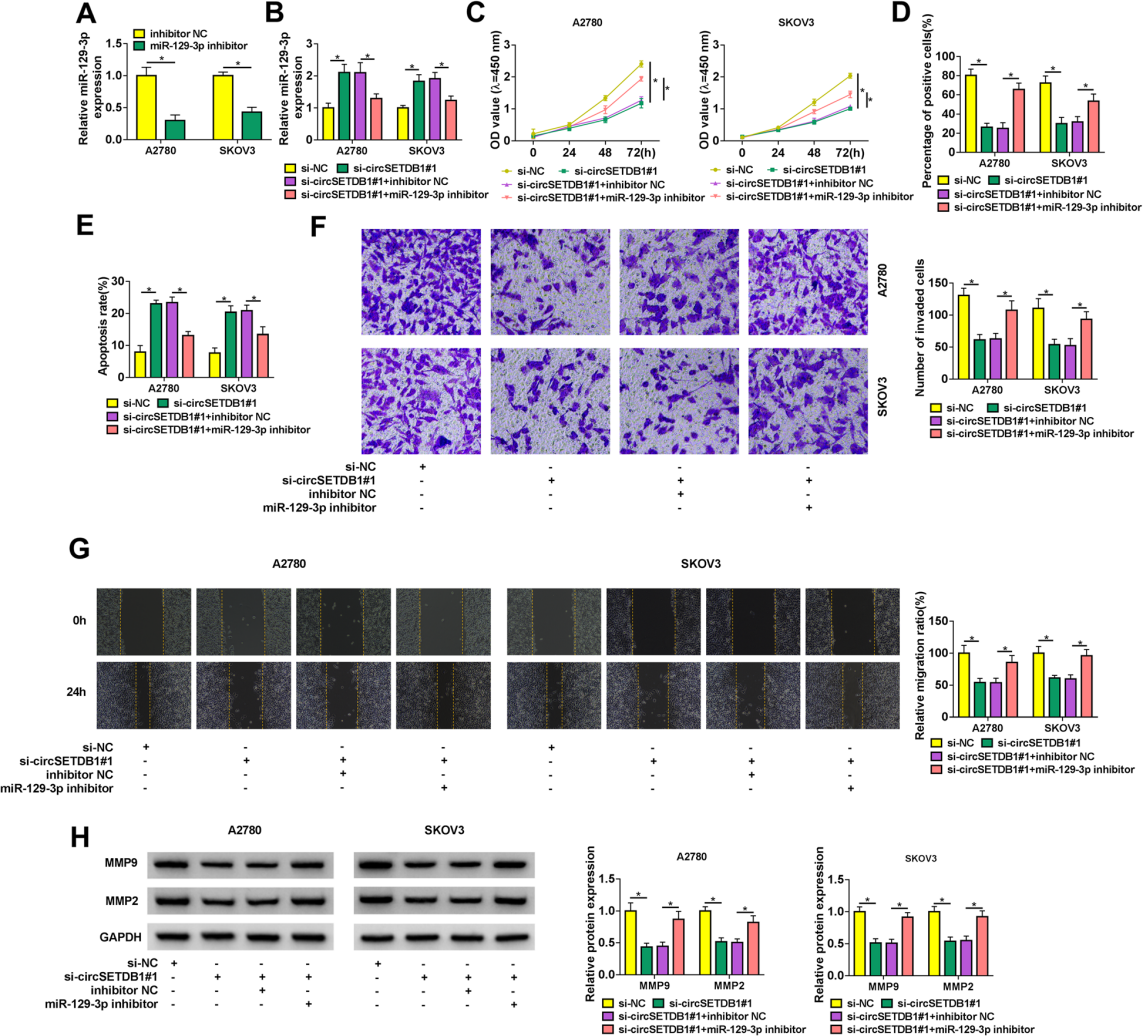
CircSETDB1 knockdown repressed SOC cell processes by interacting with miR-129-3p. **A** The interfering efficiency of miR-129-3p inhibitor was determined by qRT-PCR in A2780 and SKOV3 cells. **B**-**H** Both A2780 and SKOV3 cells were transfected with si-NC, si-circSETDB1#1, si-circSETDB1#1 + inhibitor NC or si-circSETDB1#1 + miR-129-3p inhibitor. **B** MiR-129-3p expression was detected by qRT-PCR. **C** and **D** Cell proliferation was determined by CCK-8 and EDU assays. **E** Flow cytometry analysis was performed to detect cell apoptotic rate. **F** and **G** The invasion and migration of A2780 and SKOV3 cells were investigated by transwell invasion and wound-healing assays, respectively. **H** The protein abundance of MMP9 and MMP2 was detected by western blot analysis. *P < 0.05

This study continued to reveal whether miR-129-3p was implicated in circSETDB1-mediated SOC malignant progression. The high efficiency of miR-129-3p inhibitor in reducing miR-129-3p expression was shown in Fig. 4A. Additionally, qRT-PCR data exhibited circSETDB1 silencing-caused upregulation on miR-129-3p expression was restrained by miR-129-3p inhibitor (Fig. 4B). Then, circSETDB1 knockdown repressed cell proliferation, whereas miR-129-3p inhibitor attenuated this effect (Fig. 4C and D). CircSETDB1 depletion-induced apoptosis of A2780 and SKOV3 cells was also impaired after transfection of miR-129-3p inhibitor (Fig. 4E). As expected, miR-129-3p inhibitor also reversed the effects of circSETDB1 absence on cell invasion and migration (Fig. 4F and G). Furthermore, the impacts between circSETDB1 silencing and miR-129-3p inhibitor on the protein abundance of MMP9 and MMP2 were manifested. In line with the above findings, circSETDB1 knockdown-mediated downregulation on the protein abundance of MMP9 and MMP2 was abolished by miR-129-3p inhibitors (Fig. 4H). Thus, these observations demonstrated that circSETDB1 regulated SOC progression by binding to miR-129-3p.

### CircSETDB1 knockdown decreased MAP3K3 expression through associating with miR-129-3p in A2780 and SKOV3 cells

**Fig. 5:**
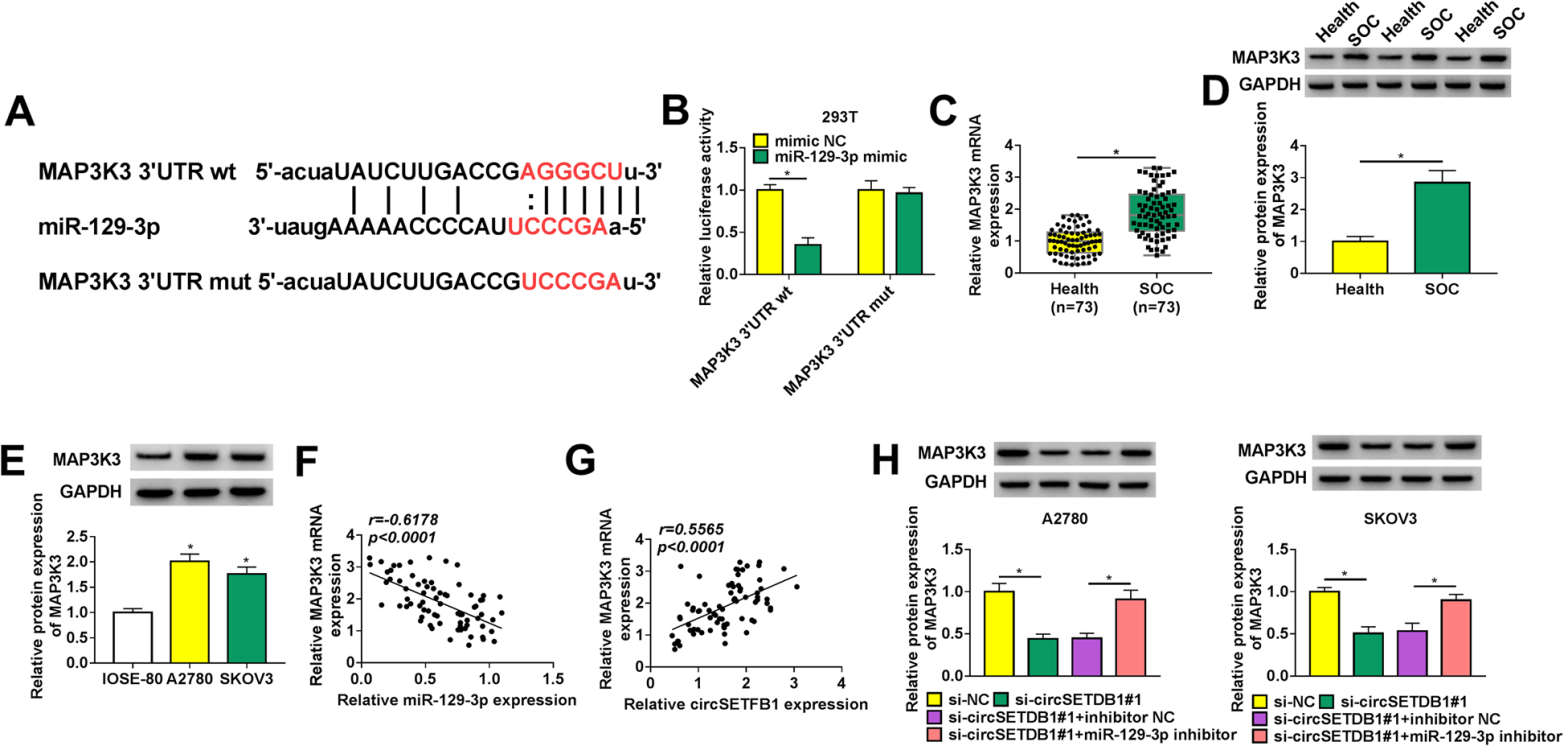
MAP3K3 expression was modulated by circSETDB1 through acting as a target gene of miR-129-3p in A2780 and SKOV3 cells. **A** The binding sequence between miR-129-3p and MAP3K3 as well as the mutated sites of MAP3K3 were shown. **B** The binding relationship between miR-129-3p and MAP3K3 was identified by dual-luciferase reporter assay in 293 T cells. **C** MAP3K3 mRNA expression was detected by qRT-PCR in 73 pairs of SOC tissues and healthy fallopian tube tissues. **D** and **E** The protein abundance of MAP3K3 was determined by western blot analysis in SOC tissues (*N* = 73), adjacent normal ovarian tissues (*N* = 73) and IOSE-80, A2780 and SKOV3 cells. **F** and **G** The correlation between MAP3K3 and miR-129-3p or circSETFB1 expression was disclosed by Spearman correlation analysis in SOC tissues. **H** The effects between circSETDB1 silencing and miR-129-3p inhibitor on MAP3K3 protein level were demonstrated by western blot analysis in A2780 and SKOV3 cells. *P < 0.05

We continued to explore the target gene of miR-129-3p. Starbase 3.0 database showed that MAP3K3 contained the binding sites of miR-129-3p (Fig. 5A), suggesting MAP3K3 might be a target gene of miR-129-3p. Subsequently, dual-luciferase reporter assay displayed that relative luciferase activity was dramatically inhibited in the co-transfection group of miR-129-3p mimic and MAP3K3 3’UTR wt, but not in the co-transfection group of miR-129-3p mimic and MAP3K3 3’UTR mut (Fig. 5B). This results demonstrated that miR-129-3p was associated with MAP3K3. Additionally, it was found that MAP3K3 mRNA and protein levels were higher in SOC tissues compared to normal fallopian tube tissues (Fig. 5C and D). A significantly high protein abundance of MAP3K3 was also found in A2780 and SKOV3 cells when compared with IOSE-80 cells (Fig. 5E). The negative correlation between miR-129-3p and MAP3K3 expression, and the positive correlation between circSETFB1 and MAP3K3 in SOC tissues were found and displayed in Fig. 5F and G. Furthermore, it was revealed using western blot analysis that circSETDB1 silencing reduced MAP3K3 protein abundance, whereas miR-129-3p inhibitor attenuated this part (Fig. 5H). These findings explained that circSETDB1 regulated MAP3K3 expression via interacting with miR-129-3p.

### MAP3K3 reintroduction attenuated miR-129-3p mimic-mediated SOC progression

**Fig. 6:**
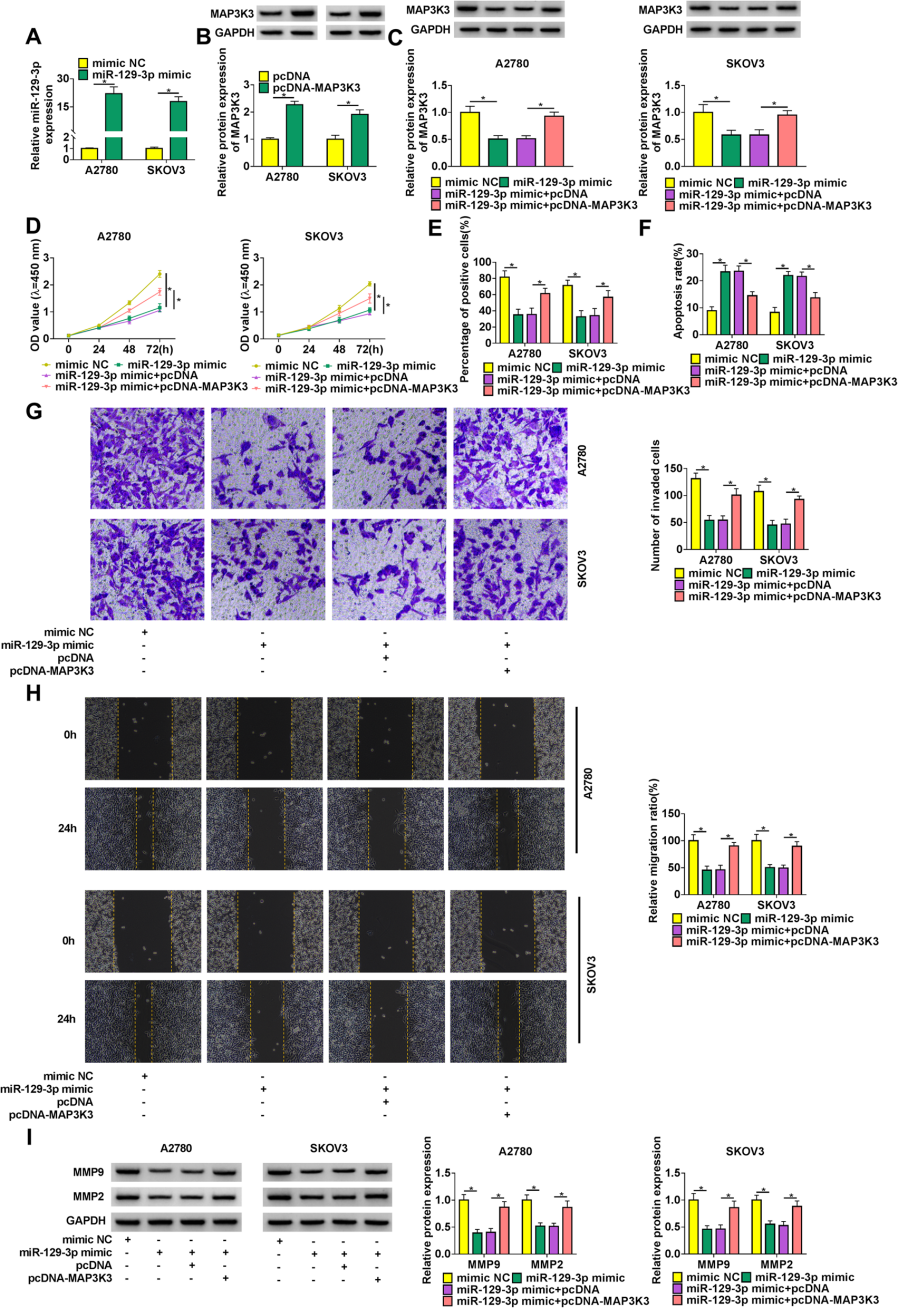
MiR-129-3p regulated SOC progression by interacting with MAP3K3. **A** MiR-129-3p expression was detected by qRT-PCR in both A2780 and SKOV3 cells transfected with miR-129-3p mimic or mimic NC. **B** Western blot analysis was performed to determine MAP3K3 protein abundance in both A2780 and SKOV3 cells transfected with pcDNA or pcDNA-MAP3K3. **C**-**I** Both A2780 and SKOV3 cells were severally transfected with mimic NC, miR-129-3p mimic, miR-129-3p mimic + pcDNA and miR-129-3p mimic + pcDNA-MAP3K3, respectively. **C** MAP3K3 protein abundance was detected by western blot analysis. **D** and **E** CCK-8 and EDU assays were conducted to investigate cell proliferation. **F** Cell apoptosis was determined by flow cytometry analysis. **G** and **H** Cell invasion and migration were revealed by transwell invasion and wound-healing assays, respectively. **I** MMP9 and MMP2 protein abundance were detected by western blot analysis. *P < 0.05

Whether miR-129-3p could regulate SOC cell processes via binding to MAP3K3 was revealed in this part. Results firstly exhibited the high efficiency of miR-129-3p mimic and pcDNA-MAP3K3 in increasing miR-129-3p or MAP3K3 expression (Fig. 6A and B). Additionally, it was found that miR-129-3p mimic decreased MAP3K3 protein abundance, whereas MAP3K3 overexpression attenuated this effect (Fig. 6C). The proliferation of A2780 and SKOV3 cells was repressed by miR-129-3p mimic, while this impact was impaired via ectopic MAP3K3 expression (Fig. 6D and E). As shown in Fig. 6F, miR-129-3p mimic-induced cell apoptosis was blocked after overexpression of MAP3K3. Also, the repressive effects of miR-129-3p mimic on the invasion and migration of A2780 and SKOV3 cells were restrained by MAP3K3 upregulation (Fig. 6G and H). MiR-129-3p mimic reduced the protein abundance of MMP9 and MMP2, which was abolished by enforced MAP3K3 expression (Fig. 6I). Furthermore, we found that reduced expression of MAP3K3 inhibited the proliferation, migration and invasion of SOC cells, but promoted their apoptosis (Supplementary Figure 1A-E). The protein levels of MMP9 and MMP2 were also decreased after MAP3K3 knockdown (Supplementary Figure 1F). To sum up, all these findings manifested that MAP3K3 participated in miR-129-3p-mediated SOC cell malignancy through functioning as a target gene of miR-129-3p.

### CircSETDB1 depletion repressed tumor formation in vivo

**Fig. 7:**
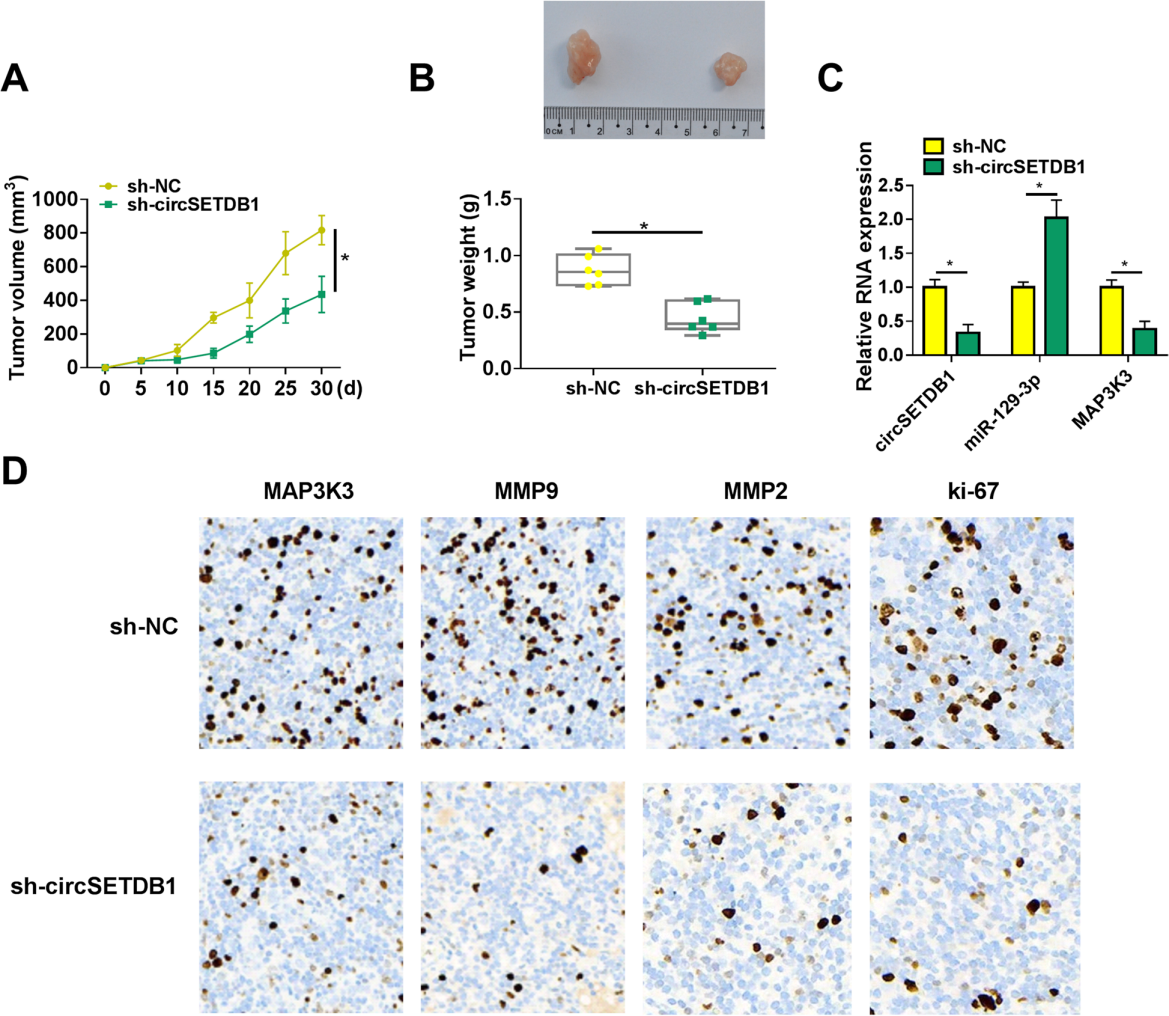
The impact of circSETDB1 on tumor formation in vivo. **A** and **B** The effects of circSETDB1 knockdown on tumor volume and weight were determined. **C** The impacts of circSETDB1 depletion on the expression of circSETDB1, miR-129-3p and MAP3K3 were determined by qRT-PCR in the neoplasms from sh-circSETDB1 group or sh-NC group. **D** IHC assay was carried out to determine the effects of circSETDB1 silencing on the positive expression rates of MAP3K3, MMP9, MMP2 and ki-67 in vivo. *P < 0.05

Given the impacts of circSETDB1 knockdown on SOC cell processes in vitro, we continued to explore the impact of circSETDB1 silencing on tumor formation in vivo. As shown in Fig. 7A and B, the volume and weight of the forming tumors were smaller or lighter in sh-circSETDB1 group than in sh-NC group, demonstrating that the downregulation of circSETDB1 restrained tumor formation. Besides, we found that circSETDB1 knockdown decreased the expression of circSETDB1 in the neoplasms (Fig. 7C), which meant that sh-circSETDB1 was effective in knocking down circSETDB1 expression in the neoplasms. The results from qRT-PCR also exhibited the increase of miR-129-3p expression and decrease of MAP3K3 expression in the neoplasms from sh-circSETDB1 group when compared with that from sh-NC group (Fig. 7C). Furthermore, the number of cells expressing MAP3K3, metastasis-related MMP9 and MMP2 or proliferation-related ki-67 were fewer in sh-circSETDB1 group than in sh-NC group (Fig. 7D), suggesting that circSETDB1 silencing repressed the protein production of MAP3K3 MMP9, MMP2 and ki-67 in vivo. Hence, all evidences manifested circSETDB1 silencing inhibited tumor formation by regulating miR-129-3p and MAP3K3 expression in vivo.

## Discussion

An increasing researches have revealed the interplay between circRNA and human disorders, including ovarian cancer [33]. Disharmony of signaling pathway and cellular mechanism serves a crucial part in cancer malignant progression [29]. The regulatory performance of circRNA in OC progression has attracted many researchers. So far, multiple investigators pay attention to the dysregulation of circRNA and its function in OC, and discover the potential of circRNA as a therapeutic agent [13]. In the present investigation, we revealed the function of circSETDB1 in SOC and underlying mechanism for the first time. In this paper, we observed high expression of circSETDB1 in SOC patients and cell lines. Moreover, we provided evidences that circSETDB1 knockdown caused the repression of proliferation and metastasis as well as the promotion of apoptosis in SOC cells. We also proved that circSETDB1 silencing restrained tumor formation by mouse model experiment. In term of mechanism, circSETDB1 induced MAP3K3 in a miR-129-3p-dependent way.

The roles of several circRNAs have been well documented in OC progression. For example, low expression of circular-phosphoglycerate mutase 1 (circ-PGAM1) produced inhibition of cell proliferation and metastasis via interaction with miR-542-3p in epithelial OC [39]. Du et al. found that circ_0015756 acted an oncogenic role in OC cell processes by the regulation in proliferative and metastatic capacities as well as apoptotic rate through miR-942-5p/Cullin 4B signaling [9]. During the study for circRNA1656, Gao and his colleagues identified that there were 710 dysregulated circRNAs in SOC specimens [12], showing the importance of circRNAs in mediating SOC progression. At present, the mechanism underlying circRNA regulated SOC development is little revealed. Our study was the first to reveal circSETDB1 role in SOC. Herein, qRT-PCR data showed high expression of circSETDB1, located in chr1:150923840–150935195 and formed from exons 14–18 of SETDB1 transcript, in SOC specimens and cell samples. CircSETDB1 was then confirmed to have higher stability than its linear parent. In addition, loss-of-function assays presented that circSETDB1 knockdown weakened cell proliferative and metastatic ability, and enhanced cell apoptotic rate in SOC cells. Furthermore, it was validated using mouse model experiment that circSETDB1 depletion repressed tumor growth and decreased the expression of metastasis-related MMP9 and MMP2 as well as proliferation-linked ki-67. Thus, these evidences showed the oncogenic role of circSETDB1 in SOC.

Considering that circRNA commonly participates in cancer progression through interacting with miRNA [8], we continued to seek circSETDB1-associated miRNA. MiR-129-3p, a candidate, has been proved to inhibit the biological functions of carious cancers. For example, in glioblastoma, Fang et al. showed that miR-129-3p reintroduction suppressed cell viability by targeting E2F transcription factor 5 [10]. In prostate cancer, miR-129-3p increase led to repression of cell proliferation and invasion by targeting SMAD family member 3 [20]. In hepatocelluar cancer, Cui and his colleagues demonstrated the repressive role of miR-129-3p in cell metastasis via binding to Aurora-A [7]. In SOC, the anticancer role of miR-129-3p was also shown by Liu and Zhao [28]. The above evidences supported miR-129-3p as the candidate of circSETDB1. In this paper, mechanism assays confirmed the interaction between miR-129-3p and circSETDB1. We also found that miR-129-3p was weakly expressed in SOC tissue and cell samples, and miR-129-3p repressed cell proliferation and metastasis. Our results were consistent with the findings from Liu and his colleagues [28]. Beyond that, miR-129-3p could be negatively regulated by circSETDB1 and induce cell apoptosis to suppress SOC malignant progression. Meanwhile, our findings also manifested miR-129-3p participated in SOC progression by interacting with circSETDB1.

Biochemical researches indicate lots of MAPKs can be activated by MAP3K3, such as ERK1/2, c-Jun N-terminal kinases (JNKs) as well as p38 [21], suggesting the importance of MAP3K3 in cancers. MAP3K3 acted as a promoter in diverse cancers, including nasopharyngeal carcinoma [38], lung cancer [42] and pancreatic cancer [32]. In this paper, based on its oncogenic role and complementary sites with miR-129-3p, MAP3K3 was also hypothesized to participate in SOC biological functions. Herein, the binding relationship between MAP3K3 and miR-129-3p was proved by dual-luciferase reporter assay. We found MAP3K3 was augmented in SOC specimen and cell samples, which was approved by the data from Jian and Dong [19]. In the report of Zhang et al. MAP3K3 displayed a promoting role in cell proliferation and metastasis of SOC [40], and we also confirmed these in this paper. Agreeing the findings of Santoro et al. [32], our results explained that MAP3K3 overexpression conferred resistance to apoptosis of SOC cells. Besides, MAP3K3 had negative correlation with miR-129-3p, and was involved in SOC development by functioning as a target gene of miR-129-3p. Given the interaction between miR-129-3p and circSETDB1 or MAP3K3, we wondered whether MAP3K3 was regulated by circSETDB1 through miR-129-3p as a bridge. Rescue experiments proved our guess that circSETDB1 silencing reduced MAP3K3 by associating with miR-129-3p.

P53 is a tumor suppressor gene and some signal transduction pathways mediated by the gene play important roles in regulating the biological behaviors of different types of cells. However, the new mechanism was confirmed with the help of p53-deficient SKOV-3 cells and the OVCAR-3 cells expressing mutant p53 in the present study. Thus, it would be interesting to explore the deep mechanism about the new findings. Given that SKOV-3 cells were resistant to several cytotoxic drugs including diphtheria toxin, cisplatin and doxorubicin, the novel mechanism might be helpful for the further study on resistance of OC to these drugs. Nevertheless, there were limitations that needed to be considered when evaluating the present study. Previous researches have revealed MAP3K3 contributes to OC growth via activating nuclear factor kappa B (NF-κB) pathway [31]. Thus, whether circSETDB1 regulates SOC progression through miR-129-3p/MAP3K3/NF-κB pathway should be testified in future. Additionally, the in vivo data about the novel mechanism is incomplete in the present study and should be explored in subsequent study.

Collectively, circSETDB1 acted as an oncogene in SOC progression by regulating cell proliferation, migration, invasion and apoptosis, and the underlying mechanism was that circSETDB1 induced MAP3K3 expression through binding to miR-129-3p. Therefore, targeting circSETDB1/miR-129-3p/MAP3K3 pathway may be a reliable strategy for OC therapy.

## Supplementary Information

**Additional file 1: Supplementary Figure 1.** MAP3K3 knockdown repressed SOC cell malignancy. (A and B) The effect of MAP3K3 knockdown on cell proliferation was revealed by CCK-8 and EDU assays in A2780 and SKOV3 cells. (C) The impact of MAP3K3 depletion on the apoptosis of A2780 and SKOV3 cells was determined by flow cytometry analysis. (D and E) Transwell invasion and wound-healing assays were employed to investigate the impacts of MAP3K3 downregulation on the invasion and migration of A2780 and SKOV3 cells, respectively. (F) Western blot analysis was performed to demonstrate the effect of MAP3K3 depletion on the protein abundance of MMP9 and MMP2 in A2780 and SKOV3 cells. *P<0.05.

## Data Availability

The analyzed data sets generated during the present study are available from the corresponding author on reasonable request.
